# Room-temperature violet luminescence and ultraviolet photodetection of Sb-doped ZnO/Al-doped ZnO homojunction array

**DOI:** 10.1186/1556-276X-8-313

**Published:** 2013-07-05

**Authors:** Wei-Jen Chen, Jen-Kai Wu, Jheng-Cyuan Lin, Shun-Tsung Lo, Huang-De Lin, Da-Ren Hang, Ming Feng Shih, Chi-Te Liang, Yuan Huei Chang

**Affiliations:** 1 Department of Physics, National Taiwan University, Taipei 106, Taiwan; 2 Graduate Institute of Applied Physics, National Taiwan University, Taipei 106, Taiwan; 3 Department of Materials and Optoelectronic Science, National Sun Yat-sen University, Kaohsiung 804, Taiwan; 4 Center for Nanoscience and Nanotechnology, National Sun Yat-sen University, Kaohsiung 804, Taiwan; 5 School of Electronic and Electrical Engineering, Sungkyunkwan University, Suwon 440-746, South Korea

**Keywords:** Violet luminescence, p-type ZnO, Photoluminescence

## Abstract

A Sb-doped ZnO microrod array was fabricated on an Al-doped ZnO thin film by electrodeposition. Strong violet luminescence, originated from free electron-to-acceptor level transitions, was identified by temperature-dependent photoluminescence measurements. This acceptor-related transition was attributed to substitution of Sb dopants for Zn sites, instead of O sites, to form a complex with two Zn vacancies (V_Zn_), the Sb_Zn_-2V_Zn_ complex. This Sb_Zn_-2V_Zn_ complex has a lower formation energy and acts as a shallow acceptor which can induce the observed strong violet luminescence. The photoresponsivity of our ZnO p-n homojunction device under a negative bias demonstrated a nearly 40-fold current gain, illustrating that our device is potentially an excellent candidate for photodetector applications in the ultraviolet wavelength region.

## Background

ZnO has gained considerable attention as a material for short-wavelength optoelectronic devices, such as light-emitting diodes [1], photodetectors [2], and laser diodes [3], because of its large bandgap (3.37 eV) and exciton binding energy (60 meV) [4,5]. As-grown ZnO is usually an n-type semiconductor because of the existence of oxygen vacancies. To enhance n-type conduction, Ga, In, or Sn can be used as extrinsic dopants. While n-doped ZnO can be readily prepared, it should be noted that p-type doping is essential for functional device applications based on ZnO. The p-type doping of ZnO is made using group V elements such as N, P, As, and Sb as dopants. Compared with n-type ZnO, the p-type ZnO is rather difficult to prepare due to the electronegative O 2*p* character of valence band maxima and the presence of n-type intrinsic defects, oxygen and Zn interstitial [6]. Therefore, the fabrication of a durable and reproducible p-type ZnO-based nanostructure remains a challenging task.

The growth of ZnO nanorod arrays has been reported using different growth methods such as pulsed laser deposition [7], thermal evaporation [8], metal-organic vapor-phase epitaxy [9], physical vapor deposition into porous anodic aluminum templates [10], or template-assisted vapor-liquid-solid and hydrothermal synthesis [11]. Recently, it was reported that ZnO nanocolumns can be grown by a low-temperature solution method [12], and arrays of vertical ZnO nanorods and nanowires grown by electrodeposition using anodic alumina [13] or polycarbonate porous membranes as templates have also been reported [14].

In this study, a Sb-doped ZnO microrod array was successfully grown on an Al-doped n-type ZnO thin film by electrodeposition. Strong violet luminescence, originated from free electron-to-acceptor level transitions, was identified by temperature-dependent photoluminescence measurements. This acceptor-related transition was attributed to substitution of Sb dopants for Zn sites, instead of O sites, to form a complex with two Zn vacancies (V_Zn_), the Sb_Zn_-2V_Zn_ complex. This Sb_Zn_-2V_Zn_ complex has a lower formation energy and acts as a shallow acceptor which can induce a strong violet luminescence. ZnO homojunction diode made with Sb-doped ZnO and Al-doped ZnO exhibits the expected p-n diode characteristic on the current-voltage (*I-V*) measurement and confirms that the Sb-doped ZnO microrod array is p-type and can be fabricated successfully using the electrodeposition method. Finally, the photoresponse of the ZnO p-n diode operating at an increased reverse bias shows a good optical response and high photocurrent gain, indicating that it can be a good candidate for use as an ultraviolet photodetector.

## Methods

A Sb-doped ZnO microrod array was electrodeposited on a patterned Al-doped n-type ZnO thin film at 98°C for 1 h [15]. The array pattern was fabricated on the ZnO thin film by optical lithography method. The reaction solution for the growth of the microrod array was a mixture of 0.05 M zinc nitrate (Zn(NO_3_)_2_·6H_2_O), 0.05 M hexamethylenetetramine (C_6_H_12_N_4_), and 0.05 g antimony acetate (Sb(CH_3_COO)_3_). The conditions for electrodeposition were optimized at *I* = 10 mA and *V* = 3.1 V. Ohmic contacts on the n-type ZnO thin film and on the Sb-doped ZnO microrod array were fabricated by depositing aluminum and gold antimony (AuSb), respectively, for the electrical characterization. The surface morphology and the crystalline structure of the sample were examined using a scanning electron microscope (SEM; HITACHI S-2400, Chiyoda-ku, Japan) and by X-ray diffraction (XRD; PANalytical X'Pert PRO, Almelo, The Netherlands), respectively. The chemical and elemental identification on the surface was carried out by X-ray photoelectron spectroscopy (XPS). A He-Cd laser at the wavelength of 325 nm was used for the photoluminescence (PL) measurement. Keithley 236 and 4200-SCS (Cleveland, OH, USA) were used for the characteristic *I-V* measurement. To study the photoresponse of our ZnO homojunction device, we employed a xenon arc lamp (LHX150 08002, Glasgow, UK) as a variable-wavelength light source. The monochromatic light was selected using an iHR-320 monochromator (HORIBA Scientific, Albany, NY, USA) and irradiated on the sample. The beam line of 365 nm was selected in the photocurrent measurement.

## Results and discussion

**Figure 1:**
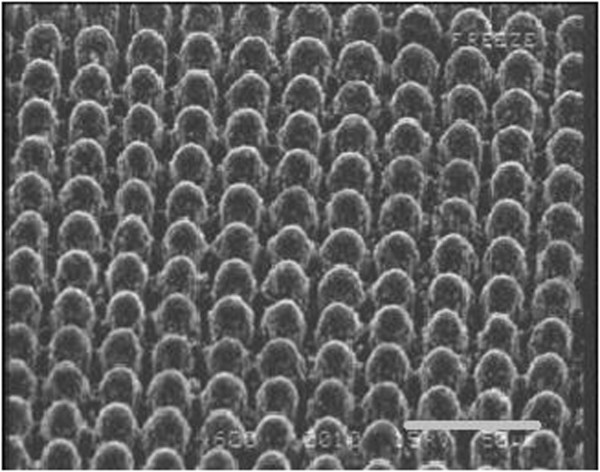
**SEM image of the Sb-doped ZnO microrod array.** The length of the scale bar is 50 μm.

**Figure 2:**
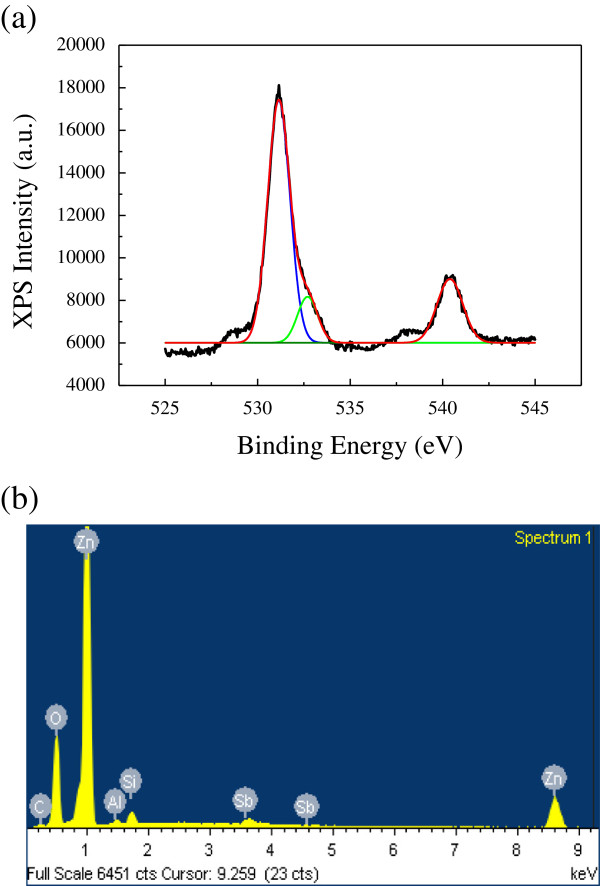
**XPS (a) and EDS (b) spectra of the Sb-doped ZnO microrod array (in bold).** The black curve shows the XPS spectrum in **(a)** while color curves display the contributions from Sb and O.

Figure 1 shows the SEM image of the Sb-doped ZnO microrod array on the n-type Al-doped ZnO substrate. From the figure, we estimate that the diameter of the ZnO microrod is about 10 μm. We can also see from this figure that the sizes of the microrods are quite uniform. Figure 2a shows the results of the XPS measurement of the Sb-doped ZnO microrod array indicated by the black curve. The peaks centered at 531.80 eV (indicated by the blue curve fit) and 540.44 eV (indicated by the red curve fit) are attributed to the binding energies of Sb_3*d*5/2_ and Sb_3*d*3/2_, which indicate the successful integration of Sb into the ZnO microrod array. The peak at 532.15 eV (indicated by the green curve fit) is attributed to O 1*s*, which mainly comes from oxygen absorption such as H_2_O, C-O, or HO- [16]. To further study the doping concentration of Sb atoms of our device, we have performed energy-dispersive X-ray spectroscopy (EDS) analysis. The measurement result presented in Figure 2b shows that the Sb concentration in the p-type ZnO is approximately 0.35%. The EDS and XPS measurements indicate clearly that antimony is incorporated into the ZnO microrods with our growth method.

**Figure 3:**
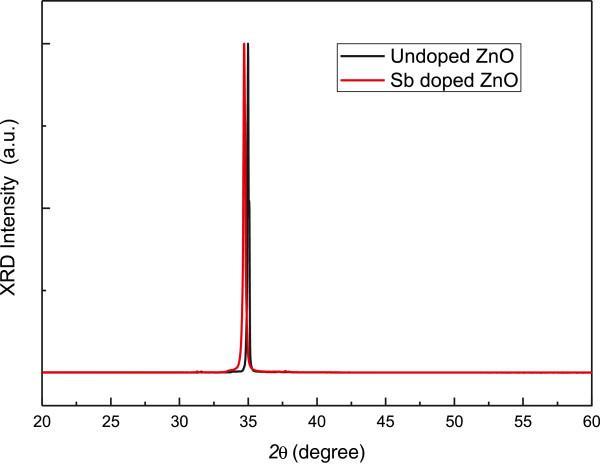
XRD data of Sb-doped ZnO and intrinsic ZnO microrod arrays.

In order to provide further studies of the Sb-doped ZnO microrod array, we have performed XRD measurements on intrinsic ZnO and Sb-doped ZnO which are shown in Figure 3. We can see in this figure that the peak of intrinsic ZnO is at 34.98° and the peak of Sb-doped ZnO is at 34.70°, which is shifted 0.28° to the left of the intrinsic peak. This peak shift can be attributed to the replacement of a Zn atom by the antimony atom introduced into the ZnO microrod array during electrodeposition and thus changes the average lattice constant [17].

**Figure 4:**
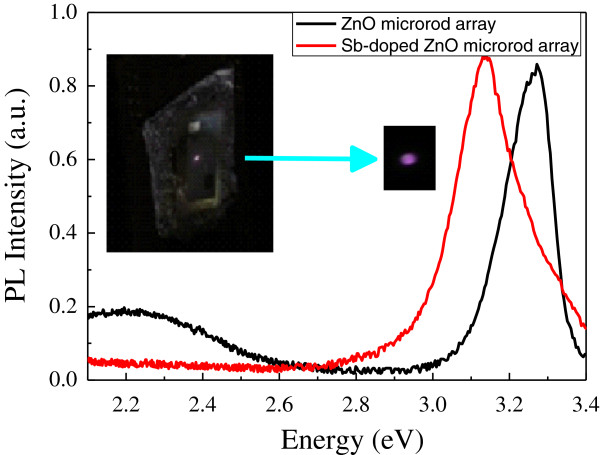
**PL measurements of Sb-doped ZnO microrod array (red) and intrinsic ZnO microrod array (black).** The inset shows a photo taken on the Sb-doped ZnO microrod array and a zoom-in showing violet luminescence.

We now turn our attention to the main finding of this paper. Figure 4 shows the PL spectra of the intrinsic ZnO and Sb-doped ZnO microrod arrays at room temperature. The intrinsic ZnO microrod array has a peak at 380 nm, corresponding to the near-band-edge peak and can be attributed to the exciton-related emission in ZnO. For the Sb-doped ZnO, we found that the PL peak shifts from the ultraviolet (380 nm) to the violet (395 nm) region of the light spectrum [18]. It is worth noting here that the yellow band that is associated with the oxygen-related defect band which shows up in the PL spectrum in the intrinsic ZnO is absent in the Sb-doped ZnO microrod array. These results suggest that the Sb-doped ZnO microrod array has a better crystalline quality. It has lower oxygen deficiencies or part of oxygen vacancies were substituted by antimony atoms; therefore, the defect band emission was reduced. Most importantly, the photo taken on the ZnO homojunction device, as shown in the inset of Figure 4, shows violet luminescence at room temperature. A zoom-in of the photo clearly shows the strong luminescence of our ZnO homojunction device.

**Figure 5:**
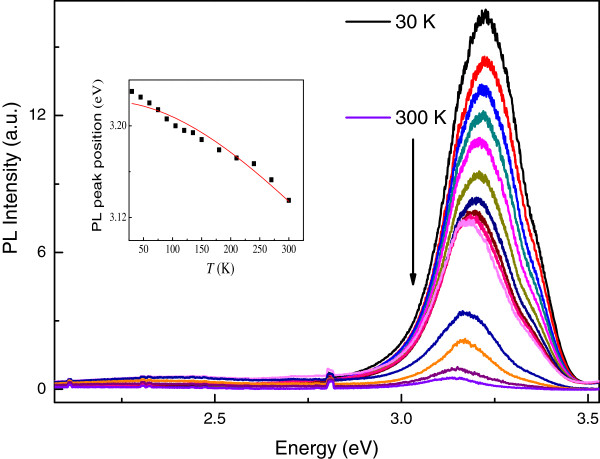
**Temperature-dependent PL spectra of the Sb-doped ZnO microrod array.** From top to bottom: *T* = 30, 45, 60, 75, 90, 105, 120, 135, 150, 180, 210, 240, 270, and 300 K, respectively. The peaks centered at around 2.8 eV are laser background signals. The inset shows the PL peak positions in energy as a function of temperature of the Sb-doped ZnO microrod array. The squares are experimental data of the FA emission, and the red line is the fitting curve to the Varshni equation.

Figure 5 shows the temperature-dependent PL spectra of the Sb-doped ZnO microrod array from *T* = 30 K to *T* = 300 K. The red shift of the PL peak along with increasing temperature can be described by the Varshni equation [19]:

(1) $$E\left(T\right)=E\left(0\right)-\alpha T^{2}/\left(\beta +T\right),$$

where *E*(0) is the transition energy of the free exciton or the free electron-to-acceptor level (FA) transition at zero temperature, and *α* and *β* are constants. The result of the fitting curve is shown in the inset of Figure 5 with *α* = 7.8 × 10^-4^ eV/K, *β* = 510 K, and *E*(0) = 3.322 eV. Moreover, the peak of the photoluminescence can be attributed to the free electron-to-acceptor level transition [16,20]. The acceptor binding energy is given by

(2) $$E_{A}=E_{g}-E_{D}-E_{\mathrm{DAP}}+\frac{e^{2}}{4\pi \epsilon \epsilon_{0}r},$$

where Eg, E_D, E_A, ϵ_0, ϵ, and r are the bandgap energy, the donor binding energy, the acceptor binding energy, the permittivity of ZnO in vacuum, the dielectric constant of ZnO, and the distance of the electron-hole pair, respectively. The donor energy ED is reported to be about 60 meV, the value of $\frac{e^{2}}{4{\pi \epsilon \epsilon }_{0}r}$ is 30 to 60 meV, and the bandgap of ZnO is 3.437 eV; therefore, the estimated EA is 161 ± 15 meV [21]. Strong violet luminescence at room temperature was revealed in this work. This particular phenomenon was induced by replacement of the Zn sites, instead of the O ones, with Sb atoms (Sb_Zn) to form a complex with two V_Zn, which is the Sb_Zn-2V_Zn complex. This Sb_Zn-2V_Zn complex has a lower formation energy and acts as a shallow acceptor; therefore, strong violet luminescence was induced as shown in Figure 5. From the room-temperature PL spectra shown in Figure 4, an estimation of the activation energy of 140 meV for Sb-doped ZnO was obtained. This value is in good agreement with the theoretical ionization energy of the Sb_Zn-2V_Zn complex acceptors [21]. The particular phenomenon has a potential application in violet light emission.

**Figure 6:**
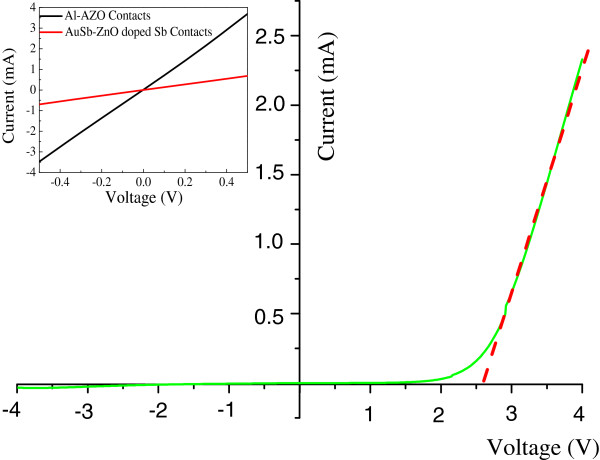
***I*-*V *measurement of the ZnO homojunction device.** The inset shows characterization of the ohmic contacts for the ZnO homojunction device.

**Figure 7:**
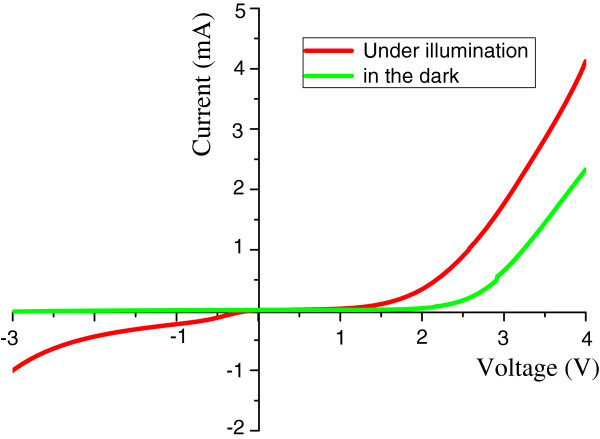
Photocurrent measurement of the ZnO homojunction device.

The *I-V* measurement of the ZnO homojunction device is shown in Figure 6. Ohmic contacts for each device were assured by the linear *I-V* relations shown in the inset of Figure 6. Therefore, the observed non-linear *I*-*V* characteristics as shown in Figure 6 must be due to the device rather than non-ideal electrical contacts. As shown in Figure 6, from the linear fit in the high-bias region (in red dashed line) [22], we can estimate the turn-on voltage of our ZnO homojunction device to be about 2.6 V, and the rectifying ratio is 24 at a voltage of 3 V. The revealed p-n junction-like *I-V* characteristic also demonstrates the successful integration of Sb in the ZnO microrod array. Figure 7 shows the measured photocurrent at various biases. At a reverse bias of -3 V, the reverse currents are 990 and 25 μA with and without the illumination of ultraviolet light, respectively. A nearly 40-fold current gain was demonstrated on this device.

**Figure 8:**
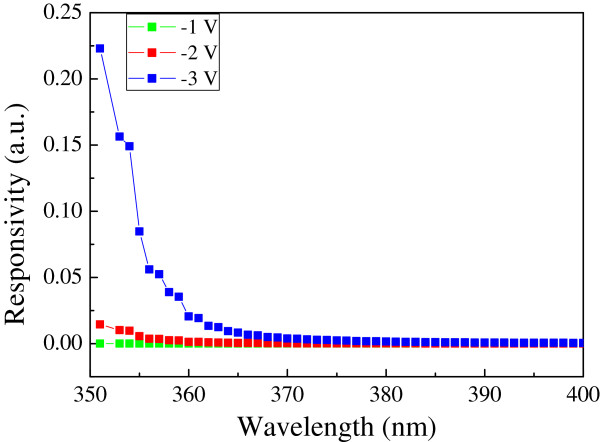
Photoresponsivity as a function of wavelength of the incident light at different reverse biases.

Finally, the photoresponsivity of the ZnO homojunction device is shown in Figure 8. At a wavelength shorter than 380 nm, the ZnO homojunction device behaves like a photodetector when a negative voltage between -1 and -3 V was applied. The responsivity of the ZnO p-n diode increases when more negative voltage was applied. Our results therefore suggest that the ZnO homojunction device has an application in photodetectors in the ultraviolet region [23,24].

## Conclusions

In this work, a high-quality Sb-doped ZnO microrod array was synthesized by electrodeposition. In Sb-doped ZnO, the shift of the XRD peak from that of the intrinsic ZnO was attributed to the increase of the lattice constant due to the replacement of a Zn atom by the Sb atom. In the case of the Sb-doped ZnO microrod array, the PL measurement indicated an acceptor-related photoemission. Strong violet luminescence at room temperature was observed since the Sb dopants would substitute Zn sites, instead of O sites, (Sb_Zn_) to form a complex with two V_Zn_, which is the Sb_Zn_-2V_Zn_ complex. This Sb_Zn_-2V_Zn_ complex has lower formation energy and acts as a shallow acceptor, which can induce a strong violet luminescence. In the *I-V* measurement, the diode-like behavior of the ZnO homojunction device indicated the successful integration of antimony atoms by electrodeposition. The nearly 40-fold current gain of the photoresponsivity of the ZnO homojunction device, acting like a p-n diode, indicates a potential application in photodetectors operating at the ultraviolet wavelength region.

## Competing interests

The authors declare that they have no competing interests.

## Authors' contributions

WJC, JKW, and JCL performed the experiments. WJC and JKW fabricated the devices. MFS and YHC coordinated the project. STL and DRH provided key interpretation of the data. WJC, HDL, DRH, and CTL drafted the paper. All authors read and approved the final manuscript.
